# Total bile acid to platelet ratio: A noninvasive index for predicting liver fibrosis in primary biliary cholangitis

**DOI:** 10.1097/MD.0000000000020502

**Published:** 2020-05-29

**Authors:** Minjie Jiang, Xin Yan, Xinyue Song, Qi Yan, Youyou Zhao, Luyuan Wang, Pujun Gao

**Affiliations:** Department of Hepatology, the First Hospital of Jilin University, Changchun, Jilin Province, China.

**Keywords:** noninvasive markers, platelet count, primary biliary cholangitis

## Abstract

The aim of the study was to develop a new early noninvasive diagnostic model for primary biliary cholangitis (PBC).

A total of 118 PBC patients who had undergone a liver biopsy were enrolled in the study, and were randomized into a model group (78 patients) and a validation group (40 patients). The patients’ histological stages were based on the classifications of the Scheuer's stage. All common parameters and liver pathological results were analyzed. And total bile acid to platelet ratio, aspartate aminotransferase to platelet ratio index, fibrosis index based on 4 factors and red cell distribution width to platelet ratio were calculated.

There were 106 (89.8%) women and 12 men in this study, and the number of patients in Scheuer stage I, II, III, and IV hepatic fibrosis was 52 (44.1%), 36 (30.5%), 26 (22.0%), and 4 (3.4%), respectively. The areas under the receiver operating characteristic curves of the total bile acid to platelet ratio (TPR), the aspartate aminotransferase to platelet ratio index, the fibrosis index based on 4 factors , and the red cell distribution width to platelet ratio for predicting advanced liver fibrosis were 0.771, 0.715, 0.618, and 0.517 respectively. The areas under the receiver operating characteristic curves of the TPR was higher than other non-invasive serological models.

As a simple, inexpensive and easily accessible non-invasive liver fibrosis diagnostic model, the TPR may be a new noninvasive marker for predicting histologic severity of PBC.

## 1 Introduction

Primary biliary cholangitis (PBC) is a progressive autoimmune cholestatic liver disease characterized by chronic cholestasis and the destruction of small and medium bile ducts. If not treated, it can develop into fibrosis, cirrhosis, or, eventually, liver failure or liver cancer.^[1]^ Typically, patients meeting 2 of the following 3 criteria are diagnosed with PBC: biochemical properties of cholestasis, presence of antimitochondrial antibody (AMA), and histologic evidence of nonsuppurative destructive cholangitis and destruction of interlobular bile ducts.^[2]^ Liver biopsy is considered the gold standard for liver fibrosis diagnosis,^[3]^ and the evaluation of histologic severity is an important determinant of PBC prognosis and survival rate.^[4]^ However, biopsies are limited by sampling error, invasiveness, cost, poor compliance, and contraindications, particularly during follow-up.^[5]^ Therefore, it is important to find inexpensive, accessible, and efficient noninvasive diagnostic methods.

Some studies found that the bile acid content is related to the pathological stage of the disease and the degree of liver fibrosis.^[6,7]^ And a study found that platelet (PLT) contribute to the reduction of liver fibrosis in mice.^[8]^ So several noninvasive methods that use laboratory indices to predict hepatic fibrosis have been studied. These noninvasive diagnostic methods include aspartat aminotransferase (AST) to platelet ratio index (APRI), fibrosis index based on 4 factors (FIB-4) score (based on AST, alanin aminotransferase (ALT), patient age, and platelet count), AST/ALT ratio (AAR), red blood cell distribution width (RDW) to platelet ratio (RPR), mean platelet volume, and cytokeratin-18.^[4,9–11]^ However they are mainly used for chronic viral liver disease and fatty liver disease. There are still few noninvasive diagnostic models for PBC because of the small number of patients with PBC. Our research aims to find a new early noninvasive diagnostic model for PBC.

## 2 Methods

### 2.1 Patients and laboratory assessment

A total of 157 PBC patients who had undergone liver biopsies between January 1, 2008 and March 31, 2018 at the First Hospital of Jilin University were enrolled in the study. The diagnostic criteria refer to the 2018 American College of Liver Diseases PBC guidelines.^[12]^ In this study, the exclusion criteria included co-infection with hepatitis virus A, hepatitis virus B, hepatitis virus C, hepatitis virus D, and/or human immunodeficiency virus, other autoimmune liver diseases, hepatocellular carcinoma, liver transplantation, and metabolic liver disease. Patients who received antiviral or anticoagulant treatment 6 months before admission were also excluded. 118 subjects were included in the final analysis.

Demographical, clinical, and laboratory data within the last 2 weeks before liver biopsy were collected. The laboratory analyses consisted of a complete blood count, including white blood cell, PLT, and platelet distribution width (PDW). Liver biochemistry analyses included AST, ALT, alkaline phosphatase (ALP), gamma glutamyl transpeptidase (GGT), cholinesterase (CHE), total bile acid (TBA), albumin, total bilirubin (TBIL), and direct bilirubin (DBIL). Other assessments included AMA, antinuclear antibody, prothrombin, and international normalized ratio.

### 2.2 Histological assessment

After receiving the patients’ informed consent, a 18G Tru-Cut needle was applied in color Doppler ultrasound-guided liver biopsy. The punctured liver tissue was required to be from 1 to 2.2 cm long and to include more than 6 complete sinks. The specimens were fixed with 10% formaldehyde solution, embedded in paraffin, and stained with hematoxylin and eosin (H&E), argentophilic staining. The pathological diagnosis for each biopsy tissue was determined using Scheuer's classification after a double-blind inspection by 2 specialists from the Pathological Diagnostic Center at the First Hospital of Jilin University. Disease stage can be categorized into 4 stages according to this histological staging system. The patients were divided into early fibrosis stage (stages I and II) and advanced fibrosis stage (stages III and IV).

### 2.3 Formulas




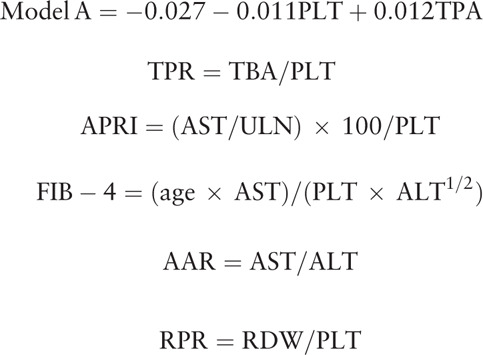




### 2.4 Statistical analysis

Measurement data were described in the form of mean ± standard deviation or median (interquartile range), and the comparison between the 2 groups was performed using an independent sample Student *t*-test or a Mann–Whitney nonparametric test. The counting data were described as a percentage, and group comparison was measured by a chi-square test. In the model group, single-factor analysis and logistic binary regression analysis were performed on each index to select independent predictors related to PBC fibrosis. The diagnostic value of total bile acid to platelet ratio (TPR), APRI, FIB-4, AAR, RPR were assessed using the area under the receiver operating curve (AUROC). *P* Values < .05 was considered statistically significant, and the statistical software used was SPSS.22.

## 3 Results

This study included 118 patients who had been diagnosed with PBC at the First Hospital of Jilin University from January 1, 2008 to March 31, 2018. There were 106 (89.8%) women and 12 men, and the number of patients in Scheuer stage I, II, III, and IV hepatic fibrosis was 52 (44.1%), 36 (30.5%), 26 (22.0%), and 4 (3.4%), respectively. The patients were randomly divided into a model group (78 cases) and a validation group (40 cases). The general information for the 2 groups is shown in Table 1. There were no statistically significant differences between the model group and validation group.

**Table 1:**
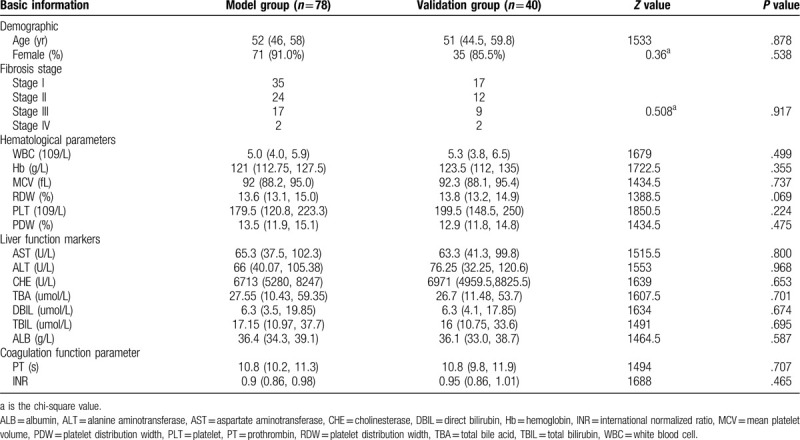
General information for the model and validation groups.

In the model group, the univariate analysis revealed differences in PLT, PDW, CHE, TBIL, TBA, and DBIL between early liver fibrosis and advanced liver fibrosis, as shown in Table 2. Compared with early liver fibrosis, patients with advanced liver fibrosis had lower PLT and CHE and higher PDW, TBA, TBIL, and DBIL. The final indicators that were entered in model-A were TBA and PLT. The regression coefficients of TBA and PLT were -0.011 and 0.012, respectively, indicating that TBA is a risk factor for advanced liver fibrosis and that PLT is a protective factor for advanced liver fibrosis. We simplified the logistic regression model named (TPR).

**Table 2:**
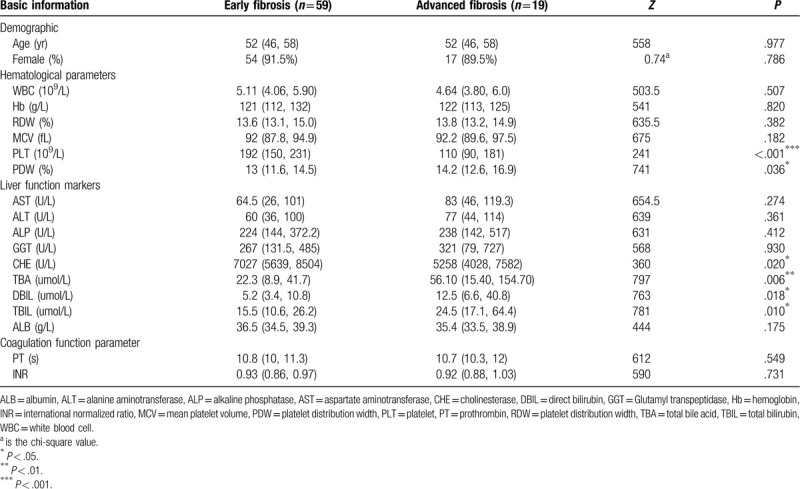
Clinical characteristics of the model group.

**Figure 1:**
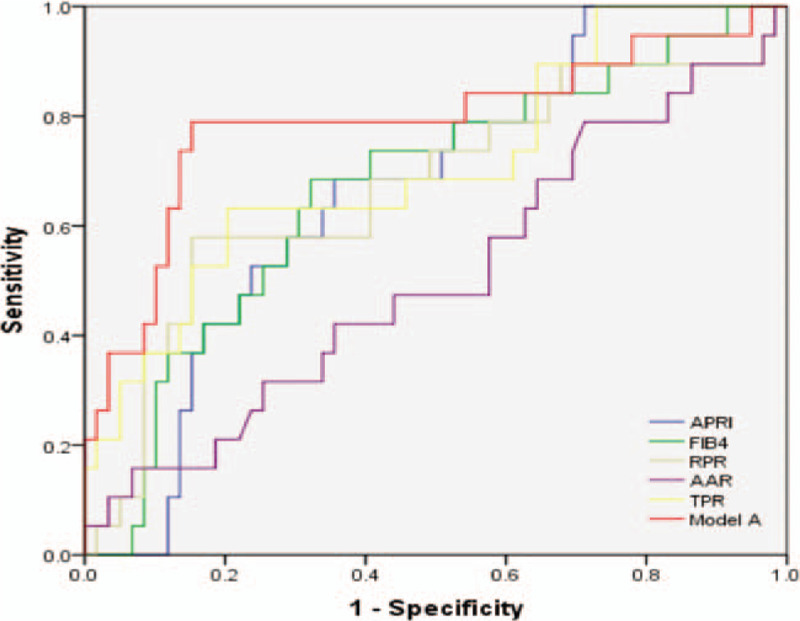
ROC curve of APRI, FIB-4, RPR, AAR, TPR, and model-A in the prediction of advanced fibrosis. TPR = total bile acid to platelet ratio, Model A = − 0.027 − 0.011PLT + 0.012TPA, APRI = aspartat aminotransferase to platelet ratio index, FIB-4 = fibrosis-4 score (based on AST, ALT, patient age, and platelet count), AAR = aspartate aminotransferase toalanine aminotransferase ratio, RPR = red blood cell distribution width to platelet ratio.

In the model group, the receiver operating characteristic curve (ROC curve) of each index is shown in Figure 1. AUROC, sensitivity, specificity, and cutoff values are shown in Table 3. The AUROCs of model A, TPR, APRI, FIB-4, and RPR for predicting liver fibrosis by ROC curve evaluation were 0.789, 0.751, 0.666, 0.670, and 0.672, respectively; model-A and the TPR had better ability to predict advanced liver fibrosis in PBC.

**Table 3:**
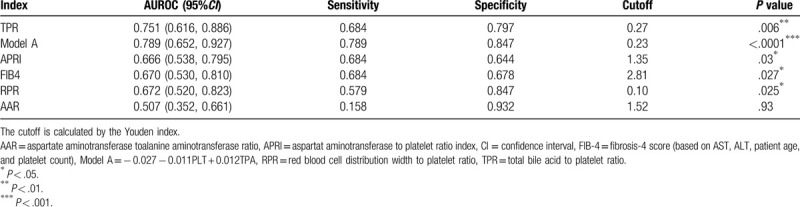
Comparison of APRI, FIB-4, RPR, AAR, TPR, and model-A for the prediction of fibrosis in advanced fibrosis.

**Figure 2:**
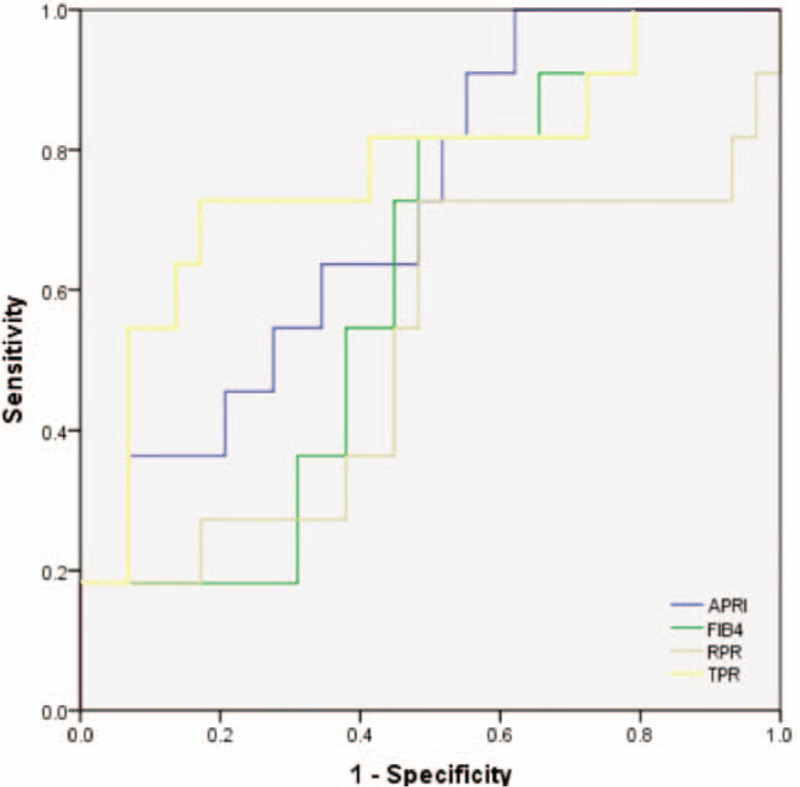
ROC curve of APRI, FIB-4, RPR, and TPR for the identification of PBC patients with advanced stage fibrosis in the validation group. TPR = total bile acid to platelet ratio, APRI = aspartat aminotransferase to platelet ratio index, FIB-4 = fibrosis-4 score (based on AST, ALT, patient age, and platelet count), RPR = red blood cell distribution width to platelet ratio.

We used a TPR index of 0.27 as the cutoff. Forty PBC patients in the validation group were evaluated for the ability of the index to predict progressive liver fibrosis. Among the 26 cases with TPR < 0.27, 23 had early liver fibrosis. Among the 14 cases with TPR > 0.27, 8 had advanced liver fibrosis. The accuracy, sensitivity, and specificity of the TPR were 77.5% (31/40), 0.727, and 0.793, respectively. After the consistency test, the kappa value of the pathological gold standard was 0.48 > 0.4, indicating that the model had good consistency with the pathological results. Among the 40 PBC patients in the validation group, the diagnostic values of TPR, APRI, AAR, FIB-4, and RPR in predicting advanced liver fibrosis were compared, and the results are shown in Table 4. The AUROCs of TPR, APRI, AAR, FIB-4, and RPR were 0.771, 0.715, 0.618, 0.517, and 0.592, respectively. Their accuracy rates were 80%, 75%, 57.4%, 65%, and 60%, respectively. The Youden indexes were 0.52, 0.203, −0.027, 0.065, and −0.117, respectively. The AUROC of the TPR was higher than that of other noninvasive serological models. The ROC curve is shown in Figure 2.

**Table 4:**
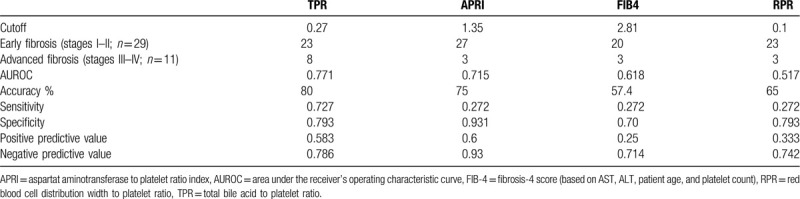
Comparison of APRI, FIB-4, RPR, and TPR for the prediction of advanced fibrosis in validation patients.

## 4 Discussion

PBC is a chronic autoimmune liver disease with long-term persistent intrahepatic cholestasis, which can eventually lead to liver cirrhosis and liver failure.^[13]^ Most patients with PBC are diagnosed with increased ALP levels and AMA positivity.^[14]^ Liver biopsy is not always necessary for the diagnosis of PBC. In AMA-negative patients with high ALP, GGT, AST, or ALT levels, liver biopsy may be needed to rule out steatohepatitis or other accompanying liver diseases.^[15]^ PBC has a good prognosis in the early stage; however, in the advanced stage of the disease, cirrhosis-related complications may occur, and so the prognosis is worse.^[16]^ Hence, histological evaluation is still useful in the pathological assessment of PBC, and to predict prognosis, but biopsies are limited by sampling error, invasiveness, cost, poor compliance, and contraindications.^[4,5,17]^ In recent years, a large number of noninvasive liver fibrosis models have emerged, including APRI, FIB-4, AAR, RPR, and FibScan.^[11,18–25]^ At present, however, they are mainly used in chronic viral liver disease and fatty liver disease, and there are still few noninvasive liver fibrosis diagnosis models for PBC.

This study found TBA to be a predictor of differentiating between early liver fibrosis and advanced liver fibrosis. The abnormal expression and/or localization of multiple transporters involved in bile acids (BAs) metabolism can be seen in the livers of PBC patients.^[26]^ The BAs content of patients with PBC increases and is closely related to disease progression. At the same time, BAs content is related to the pathological stage of the disease, ursodeoxycholic acid response, and degree of liver fibrosis.^[6,7]^ Due to elevated BAs, patients with PBC are often characterized with elevated cholestatic enzymes, such as ALP, GGT, and TBIL. It is definite that BAs assume the dispensable responsibility in PBC. This study also found that platelets are a predictor of differentiating between early and advanced liver fibrosis. The development of liver fibrosis is a complex process, and the role of platelets in the progression of fibrosis is unclear. Platelets are important for liver regeneration. Watanabe et al found that platelets contribute to the reduction of liver fibrosis in mice.^[6]^ However, platelets can also exacerbate liver damage, such as immune-mediated damage.^[24]^ Umair Iqbal et al. suggested that potent profibrogenic TGF-β and platelet-derived growth factor promote hepatic stellate cell activation and bile duct fibrosis in mice.^[27]^ A meta-analysis suggested that antiplatelet drugs have the potential to prevent the occurrence or development of liver fibrosis.^[28]^ Thus, platelets appear to have a dual role in liver fibrosis and liver cell regeneration.^[26,29,30]^All of the above mechanisms provide a theoretical basis for this study to determine whether TBA and PLT are predictors of early and progressive liver fibrosis in PBC and evaluate TPR score as a predictor of progressive liver fibrosis.

A study found RDW and RPR were related to histologic severity of PBC and its AUROC is better than APRI and FIB-4.^[20]^ However, our study did not find that RDW are related to the histological stage of PBC. At the same time, multiple studies have confirmed that many other reasons may affect RDW levels.^[31–36]^ So whether RDW can predict PBC in the advanced stage is controversial and requires large sample size verification. APRI and FIB-4 successfully predicted liver fibrosis in a large number of patients with hepatitis virus C and hepatitis virus B infections.^[37–39]^ These studies suggest that APRI and FIB-4 can be used as markers to detect the stage of moderate liver fibrosis. Therefore, they have been used in clinical practice and epidemiological research. A Japanese study found a positive correlation between APRI and histological stage of PBC.^[40]^ However, there was no statistical difference in AST between early and advanced fibrosis in this study. A possible explanation for these differences is that APRI may not be sensitive to the detection of PBC-related fibrosis. At the same time, our pathological staging system is different from the system used in Japan. In addition, the AUCROC of RPR, APRI and FIB-4 was lower than that of TPR. FibroScan test may become a simple and accurate tool for the assessment of PBC staging. However, the number of patients who received FibroScan in this study was small, so we did not analyze that data. Nevertheless, TPR scoring can be more convenient and less expensive than FibroScan.

This study has the following limitations:

(1) This was a single-center, retrospective study. There was a selection bias, and half of the patients had been treated with ursodeoxycholic acid, which affected the accuracy of the results.
(2) In our study, there were only 4 patients with histological stage IV. Therefore, this study only examined the difference between early stage fibrosis (stages I–II) and advanced stage fibrosis (stages III–IV).
(3) Due to the small number of patients who had undergone a FibroScan test, the FibroScan results were not included in this study.
(4) This model is based on patients with PBC alone, so it is not suitable for patients with overlapping AIH-PBC or other liver diseases.

In conclusion, TBA and PLT are independent predictors of liver fibrosis in the early and advanced stages of PBC. The TPR score can help distinguish between early hepatic fibrosis (Scheuer pathological stage < stage II) and advanced hepatic fibrosis (Scheuer pathological stage > II) in patients with PBC. Hence, our study findings indicated that APRI, FIB4, RPR and TPR could provide useful information for the prediction of histologic severity in PBC patients. And compared with preexisting indicators, TPR showed a higher AUROC than APRI, RPR, FIB-4, and AAR. As a result, we can use the optimal cutoff values of TPR for the diagnosis of disease severity of PBC patients. We expect to verify the efficacy of TPR score in the future with a larger sample size. At the same time, whether or not TPR score can predict the prognosis of PBC patients, evaluate the treatment response, or be applied to other types of liver disease remain subjects for further investigation.

## Author contributions

**Data curation:** Xin Yan, Xinyue Song, Qi Yan, Youyou Zhao, Luyuan Wang.

**Formal analysis:** Xin Yan.

**Writing – original draft:** Xin Yan, Pujun Gao.

**Writing – Review and Editing:** Xin Yan, Pujun Gao.

## References

[1] Al-Harthy N Kumagi T. Natural history and management of primary biliary cirrhosis. Hepatic Med Evid Res 2012;4:61–71. 10.2147/HMER.S25998 PMC3846599 24367233

[2] Lindor KD Bowlus CL Boyer J. Primary biliary cholangitis: 2018 practice guidance from the American Association for the Study of Liver Diseases. Hepatology 2019;69:394–419. 30070375 10.1002/hep.30145

[3] Zeng DW Dong J Liu YR. Noninvasive models for assessment of liver fibrosis in patients with chronic hepatitis B virus infection. World J Gastroenterol 2016;22:6663–72. 27547009 10.3748/wjg.v22.i29.6663 PMC4970475

[4] Corpechot C. Utility of noninvasive markers of fibrosis in cholestatic liver diseases. Clin Liver Dis 2016;20:143–58. 26593296 10.1016/j.cld.2015.08.013

[5] Mehta SH Lau B Afdhal NH. Exceeding the limits of liver histology markers. J Hepatol 2009;50:36–41. 19012989 10.1016/j.jhep.2008.07.039 PMC2637134

[6] Poupon RE Chrétien Y Poupon R. Serum bile acids in primary biliary cirrhosis: effect of ursodeoxycholic acid therapy. Hepatology 1993;17:599–604. 8477964 10.1002/hep.1840170412

[7] Trottier J Białek A Caron P. Metabolomic profiling of 17 bile acids in serum from patients with primary biliary cirrhosis and primary sclerosing cholangitis: a pilot study. Dig Liver Dis 2012;44:303–10. 22169272 10.1016/j.dld.2011.10.025

[8] Watanabe M Murata S Hashimoto I. Platelets contribute to the reduction of liver fibrosis in mice. J Gastroenterol Hepatol 2009;24:78–89. 18624898 10.1111/j.1440-1746.2008.05497.x

[9] Wai CT Greenson JK Fontana RJ. A simple noninvasive index can predict both significant fibrosis and cirrhosis in patients with chronic hepatitis C. Hepatology 2003;38:518–26. 12883497 10.1053/jhep.2003.50346

[10] Williams AL Hoofnagle JH. Ratio of serum aspartate to alanine aminotransferase in chronic hepatitis. Relationship to cirrhosis. Gastroenterology 1988;95:734–9. 3135226 10.1016/s0016-5085(88)80022-2

[11] Tanoglu A Sakin YS Karagöz E. Mean platelet volume may predict histological severity of primary biliary cirrhosis, but drugs and comorbidities are major concerns. Eur J Gastroenterol Hepatol 2016;28:116. 10.1097/MEG.0000000000000494 26594915

[12] Bonder A Retana A Winston DM. Prevalence of primary biliary cirrhosis-autoimmune hepatitis overlap syndrome. Clin Gastroenterol Hepatol 2011;9:609–12. 21440668 10.1016/j.cgh.2011.03.019

[13] Bowlus CL Gershwin ME. The diagnosis of primary biliary cirrhosis. Autoimmun Rev 2014;13:441–4. 24424173 10.1016/j.autrev.2014.01.041 PMC3987745

[14] European Association for the Study of the Liver. EASL Clinical Practice Guidelines: management of cholestatic liver diseases. J Hepatol 2009;51:237–67. 19501929 10.1016/j.jhep.2009.04.009

[15] Carey EJ Ali AH Lindor KD. Primary biliary cirrhosis. Lancet 2015;386:1565–75. 26364546 10.1016/S0140-6736(15)00154-3

[16] Lammers WJ Kowdley KV van Buuren HR. Predicting outcome in primary biliary cirrhosis. Ann Hepatol 2014;13:316–26. 24927602

[17] Schlichting P Hølund B Poulsen H. Liver biopsy in chronic aggressive hepatitis. Diagnostic reproducibility in relation to size of specimen. Scand J Gastroenterol 1983;18:27–32. 6675176 10.3109/00365528309181554

[18] Sterling RK Lissen E Clumeck N. Development of a simple noninvasive index to predict significant fibrosis in patients with HIV/HCV coinfection. Hepatology 2006;43:1317–25. 16729309 10.1002/hep.21178

[19] Sheth SG Flamm SL Gordon FD. AST/ALT ratio predicts cirrhosis in patients with chronic *hepatitis C virus* infection. Am J Gastroenterol 1998;93:44–8. 9448172 10.1111/j.1572-0241.1998.044_c.x

[20] Wang H Xu H Wang X. Red blood cell distribution width to platelet ratio is related to histologic severity of primary biliary cirrhosis. Medicine 2016;95:e3114. 26986159 10.1097/MD.0000000000003114 PMC4839940

[21] Sekiguchi T Umemura T Fujimori N. Serum cell death biomarkers for prediction of liver fibrosis and poor prognosis in primary biliary cirrhosis. PLOS One 2015;106:e0131658. 10.1371/journal.pone.0131658 PMC4482393 26110613

[22] Färkkilä M Rautiainen H Kärkkäinen P. Serological markers for monitoring disease progression in noncirrhotic primary biliary cirrhosis on ursodeoxycholic acid therapy. Liver Int 2008;28:787–97. 18397236 10.1111/j.1478-3231.2008.01722.x

[23] Corpechot C Gaouar F El Naggar A. Baseline values and changes in liver stiffness measured by transient elastography are associated with severity of fibrosis and outcomes of patients with primary sclerosing cholangitis. Gastroenterology 2014;1464:970–9. 10.1053/j.gastro.2013.12.030 24389304

[24] Wang L Gershwin ME Wang FS. Primary biliary cholangitis in China. Curr Opin Gastroenterol 2016;32:195–203. 26885951 10.1097/MOG.0000000000000257

[25] Hu Z Sun Y Wang Q. Red blood cell distribution width is a potential prognostic index for liver disease. Clin Chem Lab Med 2013;51:1403–8. 23314558 10.1515/cclm-2012-0704

[26] Yang H Duan Z. Bile a cids and the potential role in primary biliary cirrhosis. Digestion 2016;94:145–53. 27832649 10.1159/000452300

[27] Iqbal U Dennis BB Li AA. Use of anti-platelet agents in the prevention of hepatic fibrosis in patients at risk for chronic liver disease: a systematic review and meta-analysis. Hepatol Int 2019;13:84–90. 30539518 10.1007/s12072-018-9918-2 PMC6675411

[28] Sitia G Aiolfi R Di Lucia P. Antiplatelet therapy prevents hepatocellular carcinoma and improves survival in a mouse model of chronic hepatitis B. Proc Natl Acad Sci U S A 2012;109:E2165–72. 22753481 10.1073/pnas.1209182109 PMC3420192

[29] Yoshida S Ikenaga N Liu SB. Extrahepatic platelet-derived growth factor-β, delivered by platelets, promotes activation of hepatic stellate cells and biliary fibrosis in mice. Gastroenterology 2014;147:1378–92. 25173753 10.1053/j.gastro.2014.08.038

[30] Jacob DA Bahra M Schmidt SC. Mayo risk score for primary biliary cirrhosis: a useful tool for the prediction of course after liver transplantation? Ann Transplant 2008;13:35–42. 18806733

[31] Ma J-l Wang R Zhang FK. A noninvasive diagnostic model of liver fibrosis using serum markers in primary biliary cirrhosis. Zhonghua nei ke za zhi 2012;51:618–22. 23158860

[32] Zhang Q Cao X Zhou J. Red cell distribution width reflects the early stage residual renal function in peritoneal dialysis patients. Saudi J Kidney Dis Transpl 2018;29:1082–91. 30381504 10.4103/1319-2442.243950

[33] Yilmaz EM Kandemir A. Significance of red blood cell distribution width and C-reactive protein/albumin levels in predicting prognosis of acute pancreatitis. Ulus Travma Acil Cerrahi Derg 2018;24:528–31. 30516251 10.5505/tjtes.2018.98583

[34] Sunman H Çimen T Erat M. Red blood cell distribution width as a predictor of long-term mortality in patients with carbon monoxide poisoning. Turk J Emerg Med 2018;18:158–61. 30533559 10.1016/j.tjem.2018.05.003 PMC6261103

[35] Li J Yang X Ma J. Relationship of red blood cell distribution width with cancer mortality in hospital. BioMed Res Int 2018;2018:8914617. 30539025 10.1155/2018/8914617 PMC6261390

[36] Fickert P Wagner M. Biliary bile acids in hepatobiliary injury—What is the link? J Hepatol 2017;67:619–31. 28712691 10.1016/j.jhep.2017.04.026

[37] Trivedi PJ Bruns T Cheung A. Optimising risk stratification in primary biliary cirrhosis: AST/platelet ratio index predicts outcome independent of ursodeoxycholic acid response. J Hepatol 2014;60:1249–58. 24548531 10.1016/j.jhep.2014.01.029

[38] Teshale E Lu M Rupp LB. Apri and FIB–4 are good predictors of the stage of liver fibrosis in chronic hepatitis B: the Chronic Hepatitis Cohort Study (CHeCS). J Viral Hepat 2014;21:917–20. 25131445 10.1111/jvh.12279

[39] Stasi C Silvestri C Voller F. The epidemiological changes of HCV and HBV infections in the era of new antiviral therapies and the anti-HBV vaccine. J Infect Public Health 2016;9:389–95. 26148849 10.1016/j.jiph.2015.05.004

[40] Joshita S Umemura T Ota M. *AST*/platelet ratio index associates with progression to hepatic failure and correlates with histological fibrosis stage in Japanese patients with primary biliary cirrhosis. J Hepatol 2014;61:1443–5. 25152209 10.1016/j.jhep.2014.07.036

